# Transforming Healthcare: The AI Revolution in the Comprehensive Care of Hypertension

**DOI:** 10.3390/clinpract14040109

**Published:** 2024-07-10

**Authors:** Sreyoshi F. Alam, Maria L. Gonzalez Suarez

**Affiliations:** Nephrology and Hypertension, Mayo Clinic, Rochester, MN 55905, USA

**Keywords:** artificial intelligence, deep learning, machine learning, hypertension

## Abstract

This review explores the transformative role of artificial intelligence (AI) in hypertension care, summarizing and analyzing published works from the last three years in this field. Hypertension contributes to a significant healthcare burden both at an individual and global level. We focus on five key areas: risk prediction, diagnosis, education, monitoring, and management of hypertension, supplemented with a brief look into the works on hypertensive disease of pregnancy. For each area, we discuss the advantages and disadvantages of integrating AI. While AI, in its current rudimentary form, cannot replace sound clinical judgment, it can still enhance faster diagnosis, education, prevention, and management. The integration of AI in healthcare is poised to revolutionize hypertension care, although careful implementation and ongoing research are essential to mitigate risks.

## 1. Introduction

Hypertension and its comorbidities contribute to a large healthcare burden both at an individual and global level and there are significant challenges in its management. Diagnosis of hypertension at the physician’s office has multiple limiting factors, including the anxiety and stress associated with being in the office, time of day, etc. While there are tools such as 24 h ambulatory blood pressure monitoring (ABPM), this can often be cumbersome to the patient and may also not be readily available in all healthcare facilities. It also does not provide physicians with a tool to monitor hypertensive patients who are already on treatment. Other disadvantages of ABPM include its higher cost compared to in-office BP measurements and limitations of use with physical activity and cardiac arrhythmias [1]. Considering these issues, and with rapid advancements in Artificial Intelligence (AI) technology, scientists have looked to integrate the use of AI into the early diagnosis of hypertension, thereby preventing long-term comorbidities and complications. This is an important field where collaborations between physicians, engineers, and scientists are necessary to yield technological advancements that benefit physicians and patients alike.

**Figure 1:**
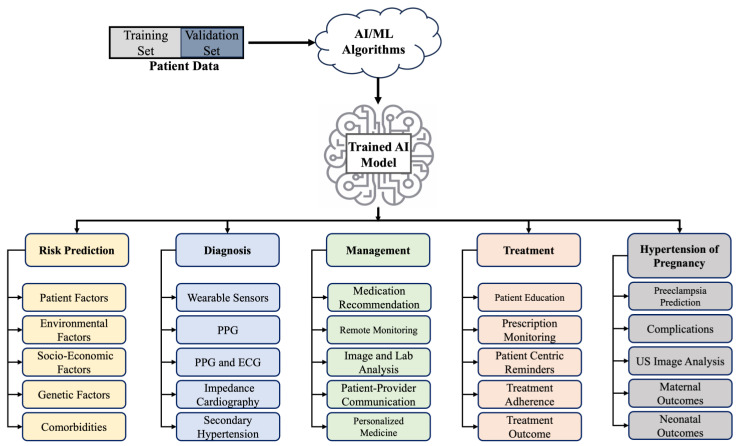
Summary of the applications of AI in the risk prediction, diagnosis, management, and treatment of hypertension [2,3,4,5,6,7,8,9,10,11,12,13,14,15,16,17,18,19,20,21,22,23,24,25,26,27,28,29,30,31,32,33,34,35,36,37,38,39,40,41,42,43].

Artificial Intelligence (AI), in simple terms, is the ability of machines to simulate human intelligence [1]. The term is often used interchangeably with machine learning (ML), although the latter is a subset of AI. Padmanabhan et al. [1] highlighted the distinction between old-fashioned AI compared to an AI that has the ability to learn from inputs, and this can be further subclassified into supervised, unsupervised, reinforced, and deep learning. The utilization of AI has taken over a myriad of aspects of everyday life. Over the last decade, we have seen some drastic changes in its use in the field of hypertension. There have already been multiple attempts at the utilization of AI in the prediction, diagnosis, and management of hypertension. Exploring 3 years of prior work, this article presents a concise overview of the advancements in the field of hypertension and the role of AI in risk prediction, early diagnosis and prevention, education, monitoring, and the management of hypertension, complemented with a brief overview of the use of AI in pregnancy-associated hypertension and pre-eclampsia. Section 1 provides a brief overview of the benefits and motivation behind the use of AI in hypertension, Section 2 articulates the published works on the use of AI in risk prediction, diagnosis, monitoring, management, treatment, and education followed by an overview of pregnancy-associated hypertension, and Section 3 concludes the paper by discussing the potential and limitations of the use of AI in hypertension care. A visual summary is provided that lists the applications of AI in risk prediction, diagnosis, management, and treatment of hypertension (Figure 1).

## 2. Methods

This paper conducts a literature review (Pubmed and Google Scholar) on research published in the last 3 years (2021–2023), highlighting the advances in the use of AI in the comprehensive care of hypertension. Our search yielded more than 150 articles. Since the focus of our review is clinician-centered, we included articles focused on research involving patients. In the subsequent subsections, we categorically discuss the utilization of AI in the fields of (a) risk prediction, (b) diagnosis, (c) monitoring, (d) treatment, and (e) education, with a brief overview of its implementation in pregnancy-associated hypertension. We also emphasize the advantages and disadvantages of AI within each premise.

### 2.1. Use of AI in Risk Prediction

Modern ML algorithms and hardware can handle large patient datasets, paving the way for its use in the prediction of factors that correlate with the future risk of development of hypertension. Initial risk prediction models relied on cross-sectional and longitudinal data. Over time, supervised ML algorithms, and clinical and genetic data, have helped improve these models [44].

Breiman et al. [2] initiated the use of ML methods like Classification and Regression Trees (CART), later developing techniques such as Bagging and Random Forests. CART has been foundational in predictive modeling, enabling more complex methodologies. Bagging, or Bootstrap Aggregating, enhances the stability and accuracy of predictions by running multiple models on different data subsets and averaging the results, thus reducing variance and improving robustness [2].

ML focuses on creating models that can make accurate predictions utilizing either linear or non-linear correlations in the data. Historically, statistics has emphasized inference and considered predictive modeling as just one aspect of the analysis. ML combines aspects of statistics and computer science within the broader field of data science. It plays a key role in areas like big data and bioinformatics, though not all of computer science is part of data science [3]. The 1990s saw contributions from computer science with the introduction of Neural Networks, Boosting, and Support Vector Machines (SVMs). These methods help analyze the relationship between an outcome and its influencing features, such as predicting hypertension based on clinical indicators [3].

A significant feature of ML-based risk prediction models is their ability to learn from data inputs [4]. Silva et al. [2] conducted a systematic review of 21 articles published between 2018 and 2021 and was the first to focus on ML and hypertension prediction; it indicated high predictive accuracy with AUROC (Area Under the Receiver Operating Characteristic curve) ranging from 0.766 to 1.00 using algorithms like support vector machines (SVMs), XGBoost (XGB), and Random Forest (RF) [2].

Montagna et al. [5] conducted an observational study by administering questionnaires, splitting the data into 14,144 training and 9062 validation sets. The study used logistic regression (LR), a decision tree classifier (DTC), RF, a SVM, and XGB. The best performance was achieved with RF, balancing sensitivity and specificity, and achieving the highest AUC of 0.816 [5]. 

A study from China analyzed data from 4,287,407 adults using Tree-based models (CART, RF, ADABoost, and XGB), as well as an ANN (Artificial Neural Network) and NB (Naive Bayes). An ANN, which mimics human brain neurons, and NB, which uses Bayes’ rule (a mathematical formula used to update the probability of a hypothesis based on new evidence) for predictions, were also evaluated. The XGB algorithm had the best performance, with an AUC of 0.894, showing that RF and XGB are effective algorithms for risk prediction [44]. 

The Random Forest (RF) model is considered an extension of CART and uses a random selection of samples and features in the training process, ensuring no dependence between the decision trees and enabling parallel operations [44]. Random Forest (RF) is a method that combines many decision trees to make more accurate predictions [44]. It uses a technique called bagging or bootstrap aggregating, which helps make the predictions more reliable and improves the stability and accuracy of ML algorithms [5]. It involves running multiple models (like decision trees, in the case of Random Forest) on different subsets of the dataset which are created through bootstrapping (sampling with replacement). The results from these models are then averaged to produce a more robust and less overfitted model. This technique is noted for enhancing predictive accuracy and controlling overfitting, which is essential in medical applications like hypertension detection where the balance between sensitivity and specificity is crucial [5].

A study in South Asia (Bangladesh, Nepal, and India) analyzed data from 818,603 participants using algorithms like XGB, Gradient Boosting Machine (GBM), LR, and Linear Discriminant Analysis (LDA). XGB and GBM achieved the highest F1 scores (95%), indicating excellent accuracy in balancing precision and recall [6]. A study in Bangladesh used four ML algorithms (an ANN, CIDT, RF, and GB) to identify hypertension risk factors. Using two cross-validation protocols with stratified random sampling repeated 25 times, the SVMRFE-GB combination achieved the highest performance: 66.98% accuracy, 97.92% recall, 78.99% F-measure, and 0.669 AUC. The study confirmed that age and BMI are strong predictors of hypertension, consistent with findings in other countries, followed by socio-economic factors. These algorithm-based models and the results of these studies align with known medical factors [7].

A cohort from Iran (4663 records) utilized ML methods to determine if body composition indices from Bioelectrical Impedance Analysis (BIA) could predict hypertension. Significant predictors included total and regional Fat Percentage (FATP), Fat-Free Mass (FFM), Basal Metabolic Rate (BMR), and age. Higher FATP and older age were directly associated with hypertension, while higher FFM and BMR were inversely related. The most accurate methods were AutoMLP, stacking, and voting, with accuracy rates of 90%, 84%, and 83%, respectively, indicating that BIA-derived body composition is a viable predictor of hypertension [8].

Finally, Nguyen et al. [9] proposed adding a DNA methylome-based deep learning (DL) model to existing models using demographic, lifestyle, and biochemical data. This study involved 50 elderly individuals and identified significant methylation sites associated with BP measures, although the small sample size limited its utility. The DL model achieved an AUPRC of 0.65 and AUROC of 0.73 [9].

Another study analyzed data from 132 individuals (healthy, pre-hypertensive, and hypertensive) from the GEO database, evenly split by gender and aged 50–65. It identified distinct epigenetic signatures in hypertensive and pre-hypertensive patients using DNA methylation levels in peripheral blood. Using ML techniques, particularly neural networks, the base model achieved 86% accuracy with 2239 CpGs, while a refined model achieved 83% accuracy with only 22 CpGs. Another model differentiated between hypertensive and pre-hypertensive patients with 88.3% accuracy using 1120 CpGs. This method, unaffected by external factors, shows promise for personalized treatment based on DNA methylation profiles, despite challenges in model complexity and interpretation [10].

After identifying the best algorithm for assessing risk prediction and risk factors, the next step is to implement it in patient care. Liao et al. [11] introduced an interpretable model (a model whose decision-making process is transparent and easily explainable and understandable to humans) for predicting hypertension and hyperlipidemia using electronic medical records (EMR). They used ML models such as XGB, CB, and RF. The CatBoost (CB) algorithm had the best performance, achieving the lowest mean standard error (MSE) of 0.0288. CatBoost excelled in predicting five targets: systolic blood pressure (SBP), diastolic blood pressure (DBP), triglycerides (TG), mHDL, and low-density lipoprotein (LDL). Performance was evaluated using MSE and loss metrics, although they did not report an AUC, unlike most studies [11]. This demonstrates that ML can be effectively integrated into EMR to identify and flag high-risk factors for hypertension.

#### Advantages and Disadvantages of AI in Hypertension Prediction

AI helps to automate data analysis, resulting in more comprehensive, deeper, and faster insights [5]. It also opens doors to the application of causal Bayesian networks, which may help emulate randomized clinical trials in the future [4]. AI’s ability to learn from data offers significant benefits, allowing for improved prediction models and enhanced data-driven decision-making [4]. AI integration in healthcare has the potential to revolutionize patient care by providing faster and more accurate diagnoses and treatment recommendations. Additionally, in theory, AI can assist in maintaining the accuracy of prediction models through frequent recalibration to address changes in clinical practices and dataset shifts. This adaptability will be requisite to ensure that AI systems remain relevant and effective over time [4].

Despite the benefits of AI’s data-driven learning capabilities, there are notable risks. One major concern is the introduction of bias and non-standardization in predictions. As AI learns from historical data, it can perpetuate societal biases, leading to inaccurate predictions for minority populations. Overcoming this issue requires extensive representation in datasets. Ethical and legal considerations, especially regarding liability for harm, are also paramount. Additionally, the public availability of prediction algorithms poses risks of data leaks [4]. Maintaining accuracy of a prediction model requires frequent recalibration to address changes in clinical practices and dataset shifts, which might pose an issue. AI models sometimes fail to account for existing guidelines; for instance, models trained on European guidelines may not perform well when applied to US populations. This discrepancy underscores the need for human oversight to catch errors and design adaptable AI systems. The Boeing 737 Max incident exemplifies the dangers of flawed AI implementation without human override, highlighting the need for clear override mechanisms in healthcare AI to prevent harm [1].

Most studies in AI-driven healthcare rely on observational data, highlighting the necessity for more practical clinical assessments beyond such studies [4]. Technical limitations, such as overfitting and underfitting, also present challenges. Overfitting occurs when a model is too closely aligned with the training data, impairing its performance on new data. Conversely, underfitting happens when a model fails to capture the predictive capabilities of the data. Addressing these issues requires large datasets, substantial informatics expertise, and a robust validation process [1].

Furthermore, deep learning models, despite their superior performance, may lack interpretability due to their “black box” nature. This opacity makes it difficult for clinicians to understand the model’s decision-making process, necessitating more research to enhance the practicality and transparency of deep learning models in clinical settings [9].

In conclusion, while AI holds immense potential to transform healthcare data analysis, it also introduces several challenges that need careful consideration and mitigation. Addressing these issues through extensive data representation, ethical considerations, technical refinements, and enhanced interpretability will be crucial for the successful integration of AI in healthcare.

### 2.2. AI-Powered Diagnosis and Monitoring of Hypertension

Now, we move to the application of AI in diagnosis and monitoring. The last 3 years have shown a spike in publications measuring BP readings using Photoplethysmography (PPG) for BP monitoring. Studies have shown that nocturnal BP readings have high predictive values of cardiovascular disease, but our current 24 h Ambulatory Blood Pressure Measurement using cuff readings has its limitations with nocturnal blood pressure monitoring. There are multiple ways to utilize PPG data to estimate blood pressure.

One notable study by Chu et al. [12] employed a deep learning model based on a Transformer architecture to predict Arterial Blood Pressure (ABP) and oxygen saturation (SpO2) from PPG signals. This model was evaluated using data from 1732 ICU patients, making it one of the largest studies in this field. The Transformer model’s attention mechanism efficiently recognizes patterns in raw data, enhancing its ability to process sequential information like PPG signals effectively [12]. In Italy, another study utilized PPG signals processed through a wavelet-based method on de-identified data from 1080 patients, amounting to over 9.1 million observations. This study employed a combination of ML models, including Extreme Gradient Boosting (XGBoost) and Neural Networks (NNs), to estimate BP. XGBoost outperformed NNs for both systolic and diastolic BP estimation, demonstrating that XGBoost, combined with selected features, can effectively estimate BP from PPG signals, adhering to clinical standards and guidelines. This paves the way for the development of wearable PPG devices integrated with ML for BP monitoring [13]. Another innovative approach involved using dual PPG sensors in a wristwatch, placed on the palmar and dorsal sides of the wrist, along with custom-made interface sensors to detect contact pressure and skin temperature. These multichannel signals were fused using a machine learning algorithm based on the Keras framework to estimate continuous BP in real time. Tested on 18 healthy subjects with 309 datasets, the device showed mean estimation errors of 0.44 ± 6.00 mmHg for systolic BP and −0.50 ± 6.20 mmHg for diastolic BP, demonstrating good agreement with actual BP measurements [14].

Similarly, the PPG2BP-Net system used a One-dimensional Convolutional Neural Network (1D-CNN) to estimate BP from PPG signals. Trained and validated with data from 4185 subjects across 25,779 surgical cases, the model showed high accuracy for both systolic and diastolic BP values. Calibration was necessary for improved accuracy, and the mean and standard deviation of SBP and mDBP were consistently around 111–112 mmHg and 61–62 mmHg, respectively [15].

Another method for BP measurements presented by Li et al. [16] introduced a thin, soft, miniaturized system (TSMS) for continuous BP monitoring. This system combines a conformal piezoelectric sensor array, an active pressure adaptation unit, and a signal processing module with advanced ML. Encapsulated in a silicone wristband, the system processes blood pulse waveforms, calculates pulse transit time intervals, and sends data to a graphical user interface. The process involves cleaning up the signals to remove noise caused by respirations and movements. The clean signals are broken down into smaller parts to analyze specific features, such as pulse shape and timing, which are linked to blood pressure. To avoid overfitting, a simpler model was preferred over complex ones and used Extreme Gradient Boosting (XGBoost) for BP estimation. Using Extreme Gradient Boosting (XGBoost), the model could accurately predict BP over a week, with most measurements falling within a 10 mmHg error range compared to standard BP monitors on the 87 volunteers [16].

Lastly, another approach involves Impedance Cardiography (ICG), which uses electrical pulses to measure changes in blood volume in the aorta after the heart pumps blood. This method has not been widely used for BP estimation yet, but shows promise [17].

Secondary hypertension, accounting for 5–10% of hypertension cases, often goes overlooked but carries a higher risk of organ damage and cardiovascular and cerebrovascular diseases. Wu et al. [18] developed a ML model to aid physicians in diagnosing secondary hypertension. The proposed two-stage framework leverages Natural Language Processing (NLP) technology to integrate unstructured text data with numerical data, converting numerical features into natural language descriptions. The dataset, comprising 98,573 cases of diagnosed hypertension from 2013 to 2019, was processed according to Chinese hypertension guidelines and ICD codes. Data processing involved dividing the dataset into training, validation, and test sets, ensuring balanced samples for each disease. The model was benchmarked against several baselines, including Logistic Regression, Random Forest, and a team of doctors with varying experience levels. The initial diagnosis stage of the model achieved an F1 score of 0.95, nearly matching the performance of the senior physicians. The model outperformed the LSTM model and was superior to a medical intern, closely aligning with the performance of more experienced doctors. By integrating unstructured text data from Electronic Health Records (EHRs) with numerical lab data using a two-stage framework, this model addresses the challenge of missed secondary hypertension diagnosis, introducing a novel approach that combines text and numerical data [18]. This paper also highlights the integration of ML models into an electronic medical record system to help with diagnosis.

#### Advantages and Disadvantages of Diagnosis and Monitoring of Hypertension with AI

Using ML for BP estimation involves creating a mathematical model to mimic a real-world system, such as the cardiovascular system. In simple terms, ML uses a ‘loss function’ to measure how far off its BP predictions are from actual BP measurements (from a cuff-based device). The goal is to minimize this difference to make the ML predictions as close as possible to the real measurements. ML algorithms take specific inputs (heartbeat data) and process them to produce an output (a BP estimate) that should match the actual BP. Standard ML includes methods like Multiple Linear Regression, which looks for linear patterns in data, and Regression Trees, which uses a series of decision-making steps to estimate BP. Advanced models such as Convolutional Neural Networks (CNNs) and Long Short-Term Memory (LSTM) models have shown promise in BP estimation [19].

Despite the advantages, the European Society of Hypertension and other governing bodies do not currently recommend cuffless BP monitors due to concerns over accuracy and reliability. Pilz et al. [19] summarized the cuffless BP monitoring progress. Many cuffless monitors use surrogates to estimate Pulse Wave Velocity (PWV), which estimates BP by measuring how fast a pulse wave travels through the body. In simple terms, PWV is a way to understand BP by looking at the speed of blood moving in the arteries. Researchers have found a method to calculate PWV by adjusting for factors like the person’s height and Pulse Arrival Time (PAT), which is calculated by the time difference between a heartbeat on an ECG (Q or R wave) and when the pulse wave reaches a certain point on the body. Another option involves Pulse Transit Time (PTT), which is like PAT but it is the time it takes for the pulse wave to travel between two points in the body. PAT at the toe gave the most accurate correlation with control BP measurements, likely because this longer distance reduces the impact of the pre-ejection period. To calculate BP, the system assumes that the heart’s systole lasts for one-third of the heart cycle, and diastole for the remaining two-thirds.

Surrogate- and estimate-based methods, like PWV and PAT, require frequent recalibration with large datasets to maintain accuracy, and the need for baseline values adds complexity [19]. It is important to highlight and understand that the use of surrogates and estimates are likely to introduce errors and would require frequent recalibration with large datasets.

CNNs are skilled at analyzing images or multidimensional data and have been used to analyze ECG and PPG curves for BP estimation. However, they require constant retraining for each patient and have high computational demands, making them impractical for on-site, real-time computing in wearable devices. LSTMs, while suitable for processing multiple inputs over time and predicting BP from heart cycle data, also demand significant computational resources and are not yet feasible for real-time, wearable devices [19].

When using models to estimate Blood Pressure (BP) without a cuff, it is important to accurately measure how much a person’s BP changes over time. If the model does not account for these changes well, the results will not be reliable [15].

A major disadvantage in designing a ML-based BP estimation model for practical cuffless BP monitoring systems is the need for large sample sizes and the prevention of overfitting [15]. Overfitting can be prevented by using separate sets for training and testing data [15]. Additionally, these models often receive mixed signals from veins and cannot measure deeply enough under the skin (usually less than 8 mm deep) [16]. PPG requires complex data processing and specific optical setups, making it challenging to develop practical wearable devices for long-term BP monitoring. Attempts to create wearable devices with ultrasound transducers and electrodes have faced difficulties due to the need for complex, high-precision, and bulky equipment, which renders them impractical for everyday use. Additionally, tonometry, which uses a pressure sensor to measure BP by detecting arterial deformations, while simpler, struggles with stability at the skin contact point. Consequently, these sensors, whether based on piezoresistive or capacitive principles, often require frequent recalibration to maintain accurate BP readings [16]. Variations in skin color and other patient-specific factors can introduce potential bias, significantly affecting the model’s applicability to a broader population. Additionally, if the study population consists of ICU patients, the model’s applicability to other patient groups or healthy individuals requires further investigation [12]. Studies that use PPG along with ECG make the process more complicated [19]. Applying ML to large amounts of genetic data to reveal outcomes is very difficult to implement in daily clinical practice, and the correlations and causations remain unclear. This process requires sophisticated analyses due to the large volumes of data and complex relationships involved, making it impractical for routine use [10]. Furthermore, none of these devices have been approved by governing bodies for clinical use. Concerns about the black box nature of AI and data security also persist, adding to the disadvantages [9].

### 2.3. Use of AI in Hypertension Management

AI can be implemented to assist in the management of hypertension in various ways. It can help physicians choose appropriate medications using patient data, support genetically targeted therapies, and aid patients with medication adherence. This discussion focuses on the use of AI in selecting suitable medications and monitoring treatment adherence. Additionally, AI can assist in monitoring treatment outcomes and detecting adverse effects of hypertension, such as cardiac remodeling and left ventricular hypertrophy.

Recent developments in the use of AI for managing hypertension also involve a trend toward personalized medicine. Wang et al. [20] conducted a comprehensive analysis to develop a model for predicting suitable antihypertensive medication regimens for elderly hypertensive patients. They tested several models, including Random Forest (RF), a Support Vector Machine (SVM), Light Gradient Boosting Machine (LightGBM), an Artificial Neural Network (ANN), and Naive Bayes (NB), using the micro-F1 score to assess efficacy. Key features for prediction included age, blood pressure metrics, and various blood test results. The LightGBM model achieved the best prediction performance, with the highest micro-F1 score of 78.4% [20].

Now moving on to medication adherence, Korb-Savoldelli et al. [23] developed and validated a new Patient-Reported Outcome Measure (PROM) for medication adherence using ML with 218 patients, including those with hypertension. The study aimed to create and validate a PROM for medication adherence by modeling the complexity and interactions among multiple patient behaviors. This cross-sectional, single-center observational study resulted in a 5-item PROM focusing on patient, treatment, and disease dimensions. A ML-derived decision tree classified patients’ medication adherence with 70% accuracy and a Negative Predictive Value (NPV) of 93%. The high NPV helps avoid unnecessary interventions for highly adherent patients. This ML-based PROM shows good psychometric properties and practical utility in clinical settings, and can be integrated into computerized prescriber order-entry systems and smartphone tools. However, further validation with a larger and more diverse population is needed to confirm its effectiveness [23].

Furthermore, AI can be used to analyze data to predict treatment outcomes. Koren et al. [21] utilized decision trees and neural networks on electronic health record (EHR) data from over 30,000 patients to predict successful treatment outcomes, defined as achieving a blood pressure lower than 140/90 mm Hg within 90 days of starting treatment. Factors like weight, age, BMI, smoking status, and concomitant treatments were used as predictors. The study found that initial BP levels and certain concurrent treatments predicted success rates, but these findings need validation through randomized trials to address potential confounding [21].

Cardiac remodeling and left ventricular hypertrophy are consequences of hypertension. A recent study developed a ML-based score to assess cardiac remodeling in young adults with hypertension, using echocardiography images from three UK studies. Analyzing 66 variables, the model derived a normalized score for 411 participants (average age 29 ± 6 years) to differentiate between hypertensive (systolic BP ≥ 160 mmHg) and normotensive individuals (systolic BP < 120 mmHg). The score, ranging from zero (healthy) to one (diseased), showed stability in cross-validation (root mean squared deviation = 0.1 ± 0.002) and effectively distinguished between the groups (Area Under the Receiver Operating Characteristics curve = 0.98). The score decreased following a 16-week exercise intervention, correlating with intervention compliance (*p* = 0.04) and improvement in ventilatory threshold (*p* = 0.01). Although promising, the study’s focus on young adults and a single location, along with non-routine heart measurements, suggests the need for broader research to enhance applicability. This ML-based score could aid in early detection and personalized management of cardiac remodeling in hypertensive patients [22].

Another application of AI for hypertension management involves its assistance with patient–provider communication. Davoudi et al. [23] conducted a study on using Natural Language Processing (NLP) and unsupervised ML to classify patient–provider messages in a digital health setting, focusing on hypertension management. The study analyzed deidentified messages from adults enrolled in Penn Medicine’s Employee Hypertension Management Program (eHTN) via a third-party mobile app. Latent Dirichlet Allocation (LDA), an unsupervised statistical model, was used to identify topics and subtopics within these messages. While LDA successfully identified common topics, it struggled with detailed intent annotation due to the complexity and heterogeneity of the messages. The study was limited to a single dataset and focused on individual messages as the unit of analysis. It demonstrated that unsupervised learning methods like LDA can group text messages into broad categories but need more detailed intent annotation for reliable NLP-based intent classifiers. This is crucial for driving clinical actions and addressing subtopic heterogeneity in digital health communication, highlighting both the potential and challenges of applying NLP and ML to enhance patient–provider communication in managing chronic conditions like hypertension [23].

#### Advantages and Disadvantages of the Use of AI in Hypertension Management

Natural Language Processing (NLP) and ML have the potential to revolutionize hypertension management by streamlining patient–provider communication. These technologies can significantly reduce the administrative burden on healthcare providers, who often face high message volumes, contributing to clinician burnout. By efficiently triaging messages to appropriate clinical teams, NLP and ML systems can reduce the workload of healthcare personnel [23]. AI provides a comprehensive evaluation of patients by integrating clinical, demographic, biochemical, and other data types, leading to better management and testing of new drug therapies. The future of hypertension management lies in personalized medicine, supported by AI technologies that use integrated data from genomics, functional genomics, protein profiling, metabolomics, and bioinformatics. AI also plays a crucial role in prognosis, considering patient demographics, organ involvement, and comorbidities. Traditional risk scores often have limitations in specificity and sensitivity, especially for certain subgroups. AI can help stratify patients more accurately, with algorithms like XGBoost showing the best prediction performance. For secondary arterial hypertension, AI can expedite diagnosis and help distinguish between primary and secondary hypertension, which is essential for treatment. ML methods have shown promise in identifying the causes of secondary hypertension and enhancing diagnostic precision and speed [24].

Despite its numerous advantages, AI cannot replace the clinician’s role and should be viewed as a tool to enhance efficiency and quality in healthcare [24]. One significant limitation is that inaccurate or biased data can lead to incorrect predictions and recommendations. There is also a risk of over-reliance on AI, which might result in clinicians overlooking important clinical details that AI systems may miss [24]. Furthermore, integrating AI into clinical practice requires substantial resources, including time and money, to develop and maintain these systems. The complexity of AI models can make them difficult to interpret, which may hinder their acceptance by healthcare providers.

Additionally, there are concerns regarding patient privacy and data security, as AI systems often require access to large amounts of sensitive health information [24]. In conclusion, while AI offers transformative potential for hypertension management through improved patient communication, personalized treatment plans, and enhanced diagnostic accuracy, it must be implemented carefully to address its limitations and ensure it complements the expertise of healthcare professionals [24].

### 2.4. Use of AI in Hypertension Education

With the advent and incorporation of AI in search engines, its use in education for both clinicians and patients has come to light. Kassab et al. [45] evaluated the effectiveness of ChatGPT 3.5, a natural language processing tool, in offering accurate advice on hypertension management in line with the 2017 American College of Cardiology/American Heart Association and 2018 European guidelines. Thirty-five questions addressing arterial hypertension were created and asked three times to ChatGPT. The responses were reviewed and graded as accurate or inaccurate by three physicians based on the American and European hypertension guidelines. ChatGPT’s responses to 31 out of the 35 questions (88%) were considered accurate. The AI model performed well in answering questions related to blood pressure treatment differences across age, sex, and race. However, the study highlighted that the model predominantly provides responses based on American guidelines, possibly due to its training data being primarily from American sources [45].

The accuracy of AI models in answering complex medical questions is still under review. While Kassab et al. [45] focused on advice on hypertension, Miao et al. [46] utilized the ChatGPT language model to answer nephrology questions. The overall accuracy of the latest ChatGPT model was 74%, below the human examinee score of 77%. For the subset of hypertension, the accuracy was 77%, with a concordance rate of 88% [46].

A study based in Japan evaluated how ChatGPT would perform in answering clinical questions (CQs) based on the Japanese Society of Hypertension (JSH) 2019 guidelines. Accuracy was defined as the proportion of correct answers out of the total number of questions. The questions were binary, numerical, or written answers. Out of 31 questions tested, ChatGPT correctly answered 20, giving it an overall accuracy rate of 64.5%. ChatGPT was more accurate in answering CQs, with an 80% accuracy rate, compared to questions based on limited evidence, where it had a 36% accuracy rate. This difference was statistically significant. ChatGPT showed a higher accuracy rate (62%) for questions related to recommendation levels than for evidence levels (38%), though this difference was not statistically significant. The accuracy of ChatGPT did not significantly differ between questions originally written in Japanese (65% accuracy) and those translated from English to Japanese (58% accuracy). Consistency was tested by Shannon Entropy in which the same question was asked 10 times; 9 out of 21 CQs always received the same answer (zero entropy), indicating high consistency. However, 7 questions showed high variability in answers (entropy > 0.5), indicating less consistency. The inconsistency was not related to the length of the text, the strength of the evidence, or the recommendations. The overall accuracy rate of 64.5% raises questions about its reliability as a tool in clinical settings [47]. Yuichiro Yano and colleagues [48] evaluated ChatGPT to ascertain if it can provide accurate and useful information to patients regarding hypertension. This study involved 20 questions in Japanese and English. The responses were evaluated by experts in the field (hypertension/nephrology). Seventeen out of twenty were considered appropriate, and unlike the previous study, responses in English were deemed to be better than in Japanese [48].

O’Hagan et al. [49] also explored the use of ChatGPT as a tool for patient education in hypertension management. The study aimed to assess ChatGPT’s ability to provide accurate and comprehensible responses to common questions patients might have about blood pressure. They posed 15 common blood pressure questions to ChatGPT in February and April 2023, and another 15 differently worded but similar questions in May 2023, to assess performance over time and with different prompts. ChatGPT’s responses were evaluated for readability, targeting a reading grade level of 8 or lower. Credibility was assessed using JAMA benchmark criteria. Accuracy was compared against U.S. and European hypertension guidelines. The average reading age of ChatGPT’s responses was higher than the ideal level, ranging from 13.5 to 14.3 across different months. None of the responses fully met the JAMA criteria for credibility. Initially, 5 out of 15 responses in February were not aligned with guidelines, improving to 3 by April. By May, most responses were consistent with guidelines, with some lacking detail in blood pressure measurement. ChatGPT’s responses generally aligned with international guidelines but varied over time, showing its capacity to adapt and improve relevance [49].

AI tools like ChatGPT show promise in educating clinicians and patients about hypertension, demonstrating high accuracy in many instances. However, the reliability and consistency of AI-generated medical advice vary, with notable differences based on training data, guidelines used, and the language of the questions.

#### Advantages and Disadvantages of AI and Hypertension Education with NLP

The application of AI in healthcare, particularly in hypertension education, has garnered significant attention. Notably, studies in the USA on cardiovascular disease prevention have highlighted the utility of AI models like ChatGPT, albeit with some limitations [50].

ChatGPT’s ability to process and generate large volumes of text rapidly makes it a valuable tool for healthcare professionals. It can be particularly helpful in addressing evidence-based questions, which are crucial in managing conditions like hypertension [46,50]. This capability can enhance the efficiency of healthcare delivery by providing quick references and augmenting the decision-making process.

Despite its potential, ChatGPT’s application in healthcare is fraught with challenges. One significant concern is the Dunning–Kruger effect, where both providers and patients might overestimate their understanding based on AI-generated responses. The rapidly evolving nature of medical knowledge means that ChatGPT’s training data can quickly become outdated, leading to plausible yet incorrect information [50]. This is compounded by the fact that ChatGPT was not originally designed for medical use, resulting in inherent biases and limitations in its training data. Furthermore, the complexity of medical language and the lack of clear referencing in ChatGPT’s responses can impact both clarity and credibility. The risk of generating outdated or incomplete information necessitates careful validation by healthcare professionals. Future research should aim to standardize the grading of AI responses and assess their readability and actionability to ensure they meet clinical standards [22]. AI models, including ChatGPT, should not be viewed as replacements for professional medical opinions [48]. To address these limitations, future studies should involve consumer-derived questions and compare AI-generated responses with those from clinicians to evaluate accuracy and effectiveness [49]. Additionally, advancements in medicine-centric NLP models, such as bioGPT, which are trained on medical literature, offer promising alternatives for more accurate and reliable medical information dissemination [51].

While AI models offer significant advantages in the realm of hypertension education, their use must be approached with caution. The benefits of quick, evidence-based responses are tempered by concerns over outdated information, lack of clear referencing, and potential biases. Ensuring these tools are used to complement, rather than replace, professional medical advice is crucial. Ongoing research and development, including the exploration of specialized models like bioGPT [51], are essential to harness the full potential of AI in healthcare.

### 2.5. Use of AI in Pregnancy-Associated Hypertension

This section highlights the recent publications on hypertension during pregnancy and the application of AI in this field. Khodari and his team [25] recently provided a comprehensive review of AI’s role in managing pregnancy-related hypertension. Hypertensive disorders affect nearly 10% of pregnant women worldwide, including gestational hypertension, chronic hypertension, and pre-eclampsia. These disorders account for 35% of global maternal deaths within years post-pregnancy, significantly contributing to maternal and neonatal morbidity and mortality. Hypertension during pregnancy is also linked to a higher long-term risk of cardiovascular events such as heart failure, stroke, and myocardial infarction. Women with hypertension in their first pregnancy face a 1.5–2.7 times higher risk of developing heart diseases later in life compared to those with normal blood pressure levels during pregnancy [25].

AI can enhance clinicians’ decision-making processes, therapy planning, and treatment protocols. However, challenges remain in the generalizability and applicability of AI models. Future work must ensure these models are interpretable and based on accessible information. Consistent results from AI models could reduce the need for continuous screening tests. Most studies have relied on electronic medical records or omics-based data, but incorporating multiple data sources like ECG and various medical imaging modalities is necessary. Larger-scale imaging data collection, linked with patient history and clinical information, could offer deeper insights into the biological changes in multiple organs during hypertensive pregnancies. A significant issue with current AI models is training on heavily unbalanced data, leading to biases. Future research should focus on including more data from minority groups (diseased patients) to develop more generalized models that reduce training bias [25]

AI has shown promising results in predicting and managing hypertension during pregnancy. The integration of telemedicine into prenatal and postpartum care accelerated during the pandemic, allowing for better blood pressure monitoring and outpatient management. Research using ML has helped to understand risks and develop individualized care models. Public health campaigns and policy changes, such as expanding Medicaid coverage, aim to improve long-term postpartum care and transition from postpartum to primary care for women with postpartum hypertension [26].

#### 2.5.1. Pre-eclampsia Prediction

In Korea, a study of 11,006 pregnant women (4.7% with pre-eclampsia) used ML models to predict late-onset pre-eclampsia using clinical information. Significant predictors included platelet counts, BUN, creatinine, potassium, calcium, and urine proteins. The XGBoost and Random Forest (RF) models had accuracy levels of 92.3% and 97.3%, respectively, with an Area Under the Curve (AUC) of 0.924 for predicting the early occurrence of pre-eclampsia [27]. Another study involving 23,201 pregnant women in Indonesia (14.3% with pre-eclampsia) used ML models (SVMs, RF, and Neural Networks) to predict pre-eclampsia based on 17 significant demographic and clinical variables. The RF model demonstrated the highest AUC levels of 0.86, 0.76, and 0.70 through 10-fold cross-validation and external geographical and temporal splitting methods [28].

In a study of 11,152 singleton pregnant women (1.28% developed hypertension), factors like maternal age, BMI, uterine artery pulsatility index, and mean arterial pressure were used to estimate pre-eclampsia risk. The RF model with 500 trees showed high performance, with an AUC of 0.86 and an accuracy of 74.5% [29].

A prospective cohort study involving 1404 pregnant women, of whom 2.4% developed hypertension, utilized a semi-supervised learning approach [30]. This method measured the similarity of each woman’s health information using Euclidean distance (a measure of the shortest path between two data points) to learn from both complete and incomplete health data. The study tested five ML methods, finding that graph-based semi-supervised learning, using the top 11 variables, provided the best prediction performance. Significant risk factors that were identified included obesity, history of pre-eclampsia, and chronic hypertension. Key variables were maternal age, blood pressure, hemoglobin level, and BMI before pregnancy. The graph-based model outperformed traditional ML models, achieving a mean AUC of 0.89 in the training set and 0.81 in the test set, surpassing both clinical guidelines and placental growth factor as a predictive biomarker. This approach allows for more accurate early prediction of pregnancy-associated hypertension using routine clinical variables. While the study demonstrated the potential of semi-supervised learning in leveraging incomplete data, further validation with larger and more diverse cohorts is needed [30].

In the study by Zhang et al. [31] on hypertensive disorders during pregnancy, 168 women were included to compare two predictive models: one using traditional hemodynamic factors like blood pressure and another using pulse wave parameters. The pulse wave model proved more effective, offering better physiological insights into cardiovascular health than blood pressure alone. Its accuracy, measured by the Area Under the Curve (AUC), exceeded 80% after the 14th week of pregnancy, peaking between 28 to 34 weeks. Despite its promising results, the study’s limitations include a small sample size and its retrospective nature, indicating the need for larger, prospective studies, but these findings suggest that pulse wave parameters could be a valuable non-invasive tool for early detection and self-monitoring of hypertensive disorders during pregnancy [31].

Pre-eclampsia disproportionately impacts racial minorities. The research by Bennett et al. [52] introduced a Cost-Sensitive Deep Neural Network (CSDNN) that addresses data imbalances and racial disparities. This model handles imbalanced, high-dimensional, and sparse data using chi-square feature selection to identify key health and personal risk factors and a focal loss function to manage uneven data distribution. The study used large datasets from Texas, Oklahoma, and the MOMI database, encompassing hundreds of thousands of patient records, providing a clearer understanding of pre-eclampsia’s impact on different groups. Accurate prediction is vital, as false negatives can lead to high maternal morbidity and mortality, while false positives may cause unnecessary interventions. The study’s limitations include a lack of detailed clinical data in the Oklahoma and Texas datasets. Future research should focus on early- and late-onset pre-eclampsia, equitable outcomes, and extending these models to other health issues with disparities [52].

#### 2.5.2. Applications of Multiomics and Machine Learning

Multiomics approaches (genomics, proteomics, metabolomics, and transcriptomics) combined with AI techniques provide a comprehensive understanding of hypertension during pregnancy. A study using genomic single-cell transcriptome data (scRNA-seq) from the European Bioinformatics Institute identified specific genes related to early-onset pre-eclampsia, achieving an accuracy of 94.62% and an AUC of 0.99. Techniques like Tuning Relief (TURF) and XGBoost identified specific genes related to placental cell subpopulations, with dendritic cells and genes such as C1QB and C1QC playing significant roles in pre-eclampsia [32,33].

Another study in India with 203 pregnant women used metabolomics and ML to investigate pregnancy-related hypertension, identifying 20 altered metabolic pathways. XGBoost and decision trees were highly effective in predictions, with some models reaching up to 98.6% accuracy [34].

A Stanford University study involving 49 pregnant women (59% with pre-eclampsia) used a multiomics approach and ML models, specifically the Elastic Net, for early prediction of pre-eclampsia. Integrating metabolome-urine and proteome models improved predictive accuracy (AUC: 0.91), revealing new connections between immune and proteomic dynamics in pre-eclampsia [35].

#### 2.5.3. Pre-Eclampsia and Complication Risk

A retrospective study with 1647 women used ML models (XGBoost and RF) to identify those at higher risk of developing complications like HELLP syndrome, cerebral hemorrhage, placental abruption, and fetal death. The models were highly effective, with a positive predictive value of 88.6% and an AUC of 0.82 [36].

Another study followed 907 women who had pre-eclampsia, assessing the development of cardiovascular diseases over a 10-year period. The RF model was particularly effective, with high sensitivity and specificity, in predicting diseases like ischemic heart disease, cerebrovascular disease, and hypertension after pre-eclampsia [37].

A prospective cohort study aimed to predict the readmission risk after adverse complications of hypertension in pregnancy [38]. The dataset included 20,032 delivering women for training and 5823 for validation. The study found significant clinical differences across variables like maternal age, gravidity, and BMI. The best-performing AI model (XGBoost) achieved AUC levels of 0.85 and 0.81 for the training and validation sets, respectively. Key factors identified by the model included systolic blood pressure changes, administered medications, and risk indicators for pre-eclampsia and hypertension [38].

Another study focused on predicting the risk of cesarean section in women with hypertension during pregnancy. Data was obtained from the Dutch multicenter randomized controlled HYPITAT trial, involving 756 pregnant women. The researchers developed two models: one using antepartum (before birth) variables and the other including intrapartum (during labor) variables. The antepartum model reached an AUC of 0.74, while the intrapartum model achieved an AUC of 0.8. Statistically significant variables included parity, ethnicity, gestational age at delivery, use of antibiotics, and proteinuria [39,40].

Villalaín et al.’s [41] study on early-onset pre-eclampsia developed two machine learning models to predict the need for delivery within 7 days and the risk of HELLP syndrome or placental abruption, using a retrospective cohort of singleton pregnancies diagnosed between 2014 and 2020. These models, showing high negative predictive values (76% and 90%), demonstrated potential clinical utility for expectant management. Of the 215 cases, 47.9% required delivery within 7 days, with a median time-to-delivery of 8 days. The SVM model with evolutionary feature selection provided valuable predictive information, with AUC values of 0.79. The advanced models, incorporating angiogenic factors and fetal ultrasound data, achieved high negative predictive values. The study’s limitations include a reliance on retrospective data, small sample size, varying laboratory standards, and limited availability of specialized sonographers. However, its strengths included reducing bias in variable selection and consistent management approaches using angiogenic markers and ultrasound evaluations [41].

#### 2.5.4. Maternal and Neonatal Outcomes

In a 7-year retrospective study involving 1829 mothers with Hypertensive Disease of Pregnancy (HDP), ML models were developed to predict adverse maternal and neonatal outcomes [42]. These models, particularly RF for maternal outcomes and boosting trees for neonatal outcomes, demonstrated high accuracy, surpassing benchmark models. The study identified early HDP diagnosis, specific ultrasound findings, and certain blood markers as early indicators of adverse outcomes [42].

#### 2.5.5. Image Analysis in Hypertension of Pregnancy

An observational study [43] used deep Convolutional Neural Networks (CNNs) to analyze ultrasound images of the placenta, distinguishing between women with hypertension and those with healthy pregnancies by examining placental texture. The study involved 429 pregnant women, 13.5% of whom had pregnancy-associated hypertension. Among the various transfer learning methods, ResNeXt achieved the highest accuracy (71%) in identifying abnormal placental textures. This AI approach significantly improved the differentiation between healthy and hypertensive pregnancies. While traditional factors like maternal age and biomarkers such as PAPP-A and PlGF help predict pre-eclampsia, their effectiveness is limited without combination, especially for late-onset pre-eclampsia. The AI model showed better sensitivity and specificity in predicting hypertension than these traditional biomarkers. The main limitation was the small sample size, affecting the robustness of the predictive model. Nevertheless, the study’s AI approach for neonatal placental analysis shows promise for non-invasive hypertension prediction in pregnancy, warranting further research and data collection. [43].

#### 2.5.6. Advantages and Disadvantages of the Use of AI in Pregnancy-Associated Hypertension

AI has shown promising results in predicting and managing hypertension during pregnancy. It enhances clinicians’ decision-making processes, therapy planning, and treatment protocols, offering consistent results that could reduce the need for continuous screening tests [25]. The integration of telemedicine into prenatal and postpartum care, accelerated by the pandemic, has improved blood pressure monitoring and outpatient management, aiding in the transition from postpartum to primary care [26]. ML models have demonstrated high accuracy in predicting pre-eclampsia, with studies reporting accuracy levels up to 97.3% and AUC values as high as 0.924 [27,28]. AI techniques such as semi-supervised learning and multiomics approaches provide comprehensive insights into hypertension, identifying significant risk factors and improving predictive performance [30,32,33]. The use of AI models has also been effective in predicting complications and long-term cardiovascular risks, demonstrating high sensitivity and specificity [36,37]. Overall, AI offers a non-invasive, precise, and early detection method for managing hypertensive disorders during pregnancy, contributing to better maternal and neonatal outcomes.

Despite its advantages, the use of AI in pregnancy-associated hypertension faces several challenges. The generalizability and applicability of AI models are hindered by their reliance on heavily unbalanced data, which can lead to biases [25]. The small sample sizes and retrospective nature of many studies limit the robustness and reliability of the predictive models, necessitating further validation with larger, prospective cohorts [31,41]. Varying laboratory standards and the limited availability of specialized sonographers can also affect the accuracy and consistency of training data and the associated AI models themselves [41]. Additionally, the lack of detailed clinical data in some datasets, such as those from Oklahoma and Texas, poses a challenge towards accurate prediction and equitable outcomes of trained AI models [43]. Addressing these limitations requires incorporating diverse data sources, improving model interpretability, and focusing on equitable outcomes to ensure AI’s effectiveness and fairness in managing hypertensive disorders during pregnancy.

## 3. Conclusions and Future Directions

In conclusion, the integration of AI into hypertension management can be a potentially significant advancement in modern medical practice. This manuscript highlights the transformative potential of AI in various aspects of hypertension care, including risk prediction, early diagnosis, monitoring, management, and education and the recent innovations in this field. AI’s ability to analyze large datasets and identify patterns has led to numerous research efforts into the development of sophisticated models that enhance the accuracy and efficiency of hypertension care.

The sheer volume of work in this field portrays an undeniable truth: AI use in hypertension indeed shows promise. However, for now, its implementation is limited due to small sample sizes in studies, hospitalized patients as sample population, lack of external validations of models, the “black box” nature of the models, and the inherent problems of overfitting and underfitting, as well as the inherent biases secondary to historical data. A small sample size may preclude good power of a study. Most of the papers did not investigate the number needed to harm/treat, which would be of clinical significance in practice. Moreover, many of the wearable devices in prior works have not been approved by governing bodies. Additionally, the push to make AI more understandable/interpretable to humans remains a significant task to enhance the trust-worthiness of these black box models. This in turn favors more simple AI algorithms and models. However, simplifying how AI works might consequently leave out important details in the perception of hidden features within the model [24]. With a view toward addressing these limitations, medical AI is already in the pipelines, such as BioGPT and MultimedQA, which uses medical literature in its training datasets [51,53]. While there has been significant progress, there is still significant room for growth in ML to help providers predict, diagnose, and manage hypertension in the near future.

While AI cannot replace the clinical judgment of healthcare professionals, it can serve as a valuable tool to enhance the speed and accuracy of hypertension diagnosis, management, and education. Continued research and development, alongside careful implementation, will be crucial in realizing the full potential of AI in transforming hypertension care and improving patient outcomes.

## Data Availability

Data sharing is not applicable.
